# Bis{μ-2,2′-[ethane-1,2-diylbis(nitrilo­methyl­idyne)]diphenolato}bis­[(thio­cyanato)manganese(III)]

**DOI:** 10.1107/S1600536808003577

**Published:** 2008-03-14

**Authors:** Shou-Bin Wang, Kun Tang, Bao-Hua Yang, Sheng Li

**Affiliations:** a College of Chemistry and Chemical Engineering, Henan University, Kaifeng 475003, People’s Republic of China; b College of Medicine, Henan University, Kaifeng 475003, People’s Republic of China

## Abstract

The reported structure is a monoclinic polymorph of the title compound, [Mn_2_(C_16_H_14_N_2_O_2_)_2_(NCS)_2_], which has been characterized previously in an ortho­rhom­bic form. Each Mn^III^ atom is chelated by a tetra­dentate 2,2′-[ethane-1,2-diylbis(nitrilo­methyl­idyne)]diphenolate ligand and by the N atom of a thio­cyanate anion, in a square-pyramidal arrangement. The complexes form centrosymmetric dimers, with an Mn—O contact of 2.557 (3) Å *trans* to each thio­cyanate anion, completing a distorted octa­hedral coordination geometry.

## Related literature

For the ortho­rhom­bic polymorph, see: Mikuriya *et al.* (1992 ▶); Li *et al.* (1997 ▶).
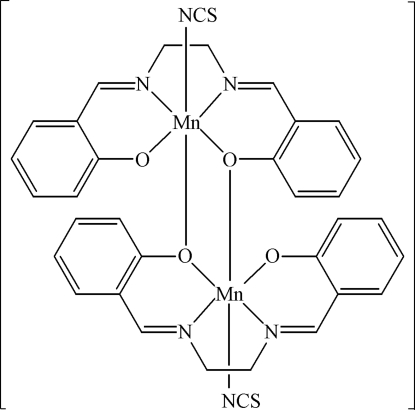


## Experimental

### 

#### Crystal data

[Mn_2_(C_16_H_14_N_2_O_2_)_2_(NCS)_2_]
*M* *_r_* = 758.62
Monoclinic, 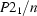

*a* = 9.0026 (10) Å
*b* = 14.0629 (16) Å
*c* = 14.9884 (17) Å
β = 106.848 (1)°
*V* = 1816.1 (4) Å^3^
*Z* = 2
Mo *K*α radiation
μ = 0.85 mm^−1^
*T* = 293 (2) K
0.43 × 0.28 × 0.22 mm

#### Data collection

Bruker APEXII CCD diffractometer
Absorption correction: multi-scan (*SADABS*; Bruker, 2001 ▶) *T*_min_ = 0.710, *T*_max_ = 0.834
13254 measured reflections
3296 independent reflections
2627 reflections with *I* > 2σ(*I*)
*R*_int_ = 0.023

#### Refinement

*R*[*F* ^2^ > 2σ(*F* ^2^)] = 0.066
*wR*(*F* ^2^) = 0.231
*S* = 1.00
3296 reflections
193 parameters
H-atom parameters constrained
Δρ_max_ = 1.47 e Å^−3^
Δρ_min_ = −0.33 e Å^−3^

### 

Data collection: *APEX2* (Bruker, 2004 ▶); cell refinement: *SAINT-Plus* (Bruker, 2001 ▶); data reduction: *SAINT-Plus*; program(s) used to solve structure: *SHELXS97* (Sheldrick, 2008 ▶); program(s) used to refine structure: *SHELXL97* (Sheldrick, 2008 ▶); molecular graphics: *SHELXTL* (Sheldrick, 2008 ▶); software used to prepare material for publication: *SHELXTL*.

## Supplementary Material

Crystal structure: contains datablocks I, global. DOI: 10.1107/S1600536808003577/bi2278sup1.cif

Structure factors: contains datablocks I. DOI: 10.1107/S1600536808003577/bi2278Isup2.hkl

Additional supplementary materials:  crystallographic information; 3D view; checkCIF report

## Figures and Tables

**Table:** 

Mn1—O1	1.874 (3)
Mn1—O2	1.902 (3)
Mn1—N1	1.979 (4)
Mn1—N2	1.990 (5)
Mn1—N3	2.181 (5)
Mn1—O2^i^	2.557 (3)

**Table:** 

O1—Mn1—O2	94.95 (14)
O1—Mn1—N1	92.1 (2)
O1—Mn1—N2	170.90 (17)
O1—Mn1—N3	93.94 (18)
O1—Mn1—O2^i^	88.62 (15)
O2—Mn1—N1	165.40 (19)
O2—Mn1—N2	89.51 (17)
O2—Mn1—N3	96.85 (17)
O2—Mn1—O2^i^	80.14 (14)
N1—Mn1—N2	81.9 (2)
N1—Mn1—N3	95.4 (2)
N1—Mn1—O2^i^	87.29 (16)
N2—Mn1—N3	93.4 (2)
N2—Mn1—O2^i^	84.35 (17)
N3—Mn1—O2^i^	176.24 (17)

Symmetry code: (i) 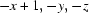.

**Fig. 1.**
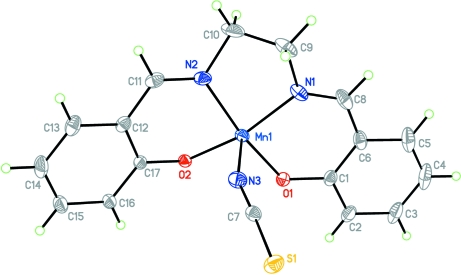
Asymmetric unit drawn with 30% probability displacement ellipsoids for the non-H atoms.

## supplementary crystallographic information

### Comment

As shown in Fig. 1, the Mn atom is chelated by two N and two O atoms of the *N*,*N*'-ethylenebis(salicylideneamine) ligand and by the N atom of the thiocyanate anion in the apical site. The Mn—N and Mn—O bond lengths are in the range of 1.979 (4)–2.181 (5) and 1.874 (3)–1.902 (3) %A, respectively (Table 1).

### Experimental

A mixture of manganese(III) acetate (1 mmol) and *N*,*N*'-bis(2-hydroxybenzyl)ethylenediamine (1 mmol) in 20 ml me thanol was refluxed for several hours. The solution was then cooled and filtered, and the filtrate was left to evaporate at room temperature. Pink blocks of the title compound were obtained after 2 days with a yield of 12%. Elemental analysis calculated: C 53.72, H 3.67, N 5.49%; found: C 53.78, H 3.69, N 5.54%.

### Refinement

H atoms were placed in calculated positions and refined as riding with *U*_iso_(H) = 1.2 *U*_eq_(C). The phenyl rings were constrained to have regular hexagonal geometry.

### Crystal data

**Table:**

[Mn_2_(C_16_H_14_N_2_O_2_)_2_(NCS)_2_]	*F*_000_ = 776
*M**_r_* = 758.62	*D*_x_ = 1.387 Mg m^−^^3^
Monoclinic, *P*2_1_/*n*	Mo *K*α radiation λ = 0.71073 Å
Hall symbol: -P 2yn	Cell parameters from 4497 reflections
*a* = 9.0026 (10) Å	θ = 2.4–24.4º
*b* = 14.0629 (16) Å	µ = 0.86 mm^−^^1^
*c* = 14.9884 (17) Å	*T* = 293 (2) K
β = 106.848 (1)º	Block, pink
*V* = 1816.1 (4) Å^3^	0.43 × 0.28 × 0.22 mm
*Z* = 2	

### Data collection

**Table:**

Bruker APEXII CCD diffractometer	3296 independent reflections
Radiation source: fine-focus sealed tube	2627 reflections with *I* > 2σ(*I*)
Monochromator: graphite	*R*_int_ = 0.023
*T* = 295(2) K	θ_max_ = 25.3º
φ and ω scans	θ_min_ = 2.4º
Absorption correction: multi-scan(SADABS; Bruker, 2001)	*h* = −10→10
*T*_min_ = 0.710, *T*_max_ = 0.834	*k* = −16→16
13254 measured reflections	*l* = −18→17

### Refinement

**Table:**

Refinement on *F*^2^	Secondary atom site location: difference Fourier map
Least-squares matrix: full	Hydrogen site location: inferred from neighbouring sites
*R*[*F*^2^ > 2σ(*F*^2^)] = 0.066	H-atom parameters constrained
*wR*(*F*^2^) = 0.231	*w* = 1/[σ^2^(*F*_o_^2^) + (0.147*P*)^2^ + 2.2875*P*] where *P* = (*F*_o_^2^ + 2*F*_c_^2^)/3
*S* = 1.00	(Δ/σ)_max_ = 0.013
3296 reflections	Δρ_max_ = 1.47 e Å^−^^3^
193 parameters	Δρ_min_ = −0.33 e Å^−^^3^
Primary atom site location: structure-invariant direct methods	Extinction correction: none

### Special details

**Table:**

Geometry. All e.s.d.'s (except the e.s.d. in the dihedral angle between two l.s. planes) are estimated using the full covariance matrix. The cell e.s.d.'s are taken into account individually in the estimation of e.s.d.'s in distances, angles and torsion angles; correlations between e.s.d.'s in cell parameters are only used when they are defined by crystal symmetry. An approximate (isotropic) treatment of cell e.s.d.'s is used for estimating e.s.d.'s involving l.s. planes.
Refinement. Refinement of *F*^2^ against ALL reflections. The weighted *R*-factor *wR* and goodness of fit *S* are based on *F*^2^, conventional *R*-factors *R* are based on *F*, with *F* set to zero for negative *F*^2^. The threshold expression of *F*^2^ > σ(*F*^2^) is used only for calculating *R*-factors(gt) *etc*. and is not relevant to the choice of reflections for refinement. *R*-factors based on *F*^2^ are statistically about twice as large as those based on *F*, and *R*- factors based on ALL data will be even larger.

### Fractional atomic coordinates and isotropic or equivalent isotropic displacement parameters (Å^2^)

**Table:**

	*x*	*y*	*z*	*U*_iso_*/*U*_eq_	
Mn1	0.64485 (8)	0.01038 (5)	0.10349 (5)	0.0575 (3)	
C1	0.8655 (4)	0.1314 (2)	0.0466 (3)	0.0705 (14)	
C2	0.9625 (5)	0.1411 (3)	−0.0101 (3)	0.093 (2)	
H2	0.9766	0.0902	−0.0465	0.112*	
C3	1.0384 (5)	0.2267 (4)	−0.0124 (4)	0.124 (3)	
H3	1.1033	0.2332	−0.0503	0.148*	
C4	1.0173 (6)	0.3027 (3)	0.0420 (4)	0.146 (4)	
H4	1.0681	0.3600	0.0404	0.175*	
C5	0.9204 (6)	0.2931 (3)	0.0987 (4)	0.128 (3)	
H5	0.9063	0.3439	0.1351	0.153*	
C6	0.8445 (5)	0.2074 (3)	0.1010 (3)	0.0848 (17)	
C7	0.9436 (7)	−0.0871 (3)	0.2500 (4)	0.0640 (12)	
C8	0.7500 (9)	0.2027 (5)	0.1635 (4)	0.098 (2)	
H8	0.7466	0.2568	0.1986	0.117*	
C9	0.5841 (12)	0.1325 (5)	0.2457 (5)	0.117 (3)	
H9A	0.5629	0.1977	0.2592	0.140*	
H9B	0.6451	0.1027	0.3030	0.140*	
C10	0.4299 (12)	0.0775 (6)	0.2049 (7)	0.125 (3)	
H10A	0.3826	0.0616	0.2535	0.150*	
H10B	0.3571	0.1147	0.1574	0.150*	
C11	0.4148 (7)	−0.0916 (5)	0.1681 (4)	0.0801 (16)	
H11	0.3420	−0.0952	0.2011	0.096*	
C12	0.4489 (4)	−0.1778 (2)	0.1251 (2)	0.0686 (13)	
C13	0.4001 (5)	−0.2635 (3)	0.1534 (3)	0.0921 (19)	
H13	0.3496	−0.2641	0.1994	0.110*	
C14	0.4266 (6)	−0.3482 (2)	0.1127 (3)	0.106 (2)	
H14	0.3939	−0.4055	0.1316	0.128*	
C15	0.5020 (5)	−0.34726 (18)	0.0439 (3)	0.0937 (19)	
H15	0.5197	−0.4040	0.0167	0.112*	
C16	0.5508 (4)	−0.2616 (2)	0.0156 (2)	0.0723 (14)	
H16	0.6013	−0.2610	−0.0304	0.087*	
C17	0.5243 (4)	−0.17685 (18)	0.0563 (2)	0.0592 (11)	
N1	0.6693 (7)	0.1302 (3)	0.1756 (3)	0.0799 (13)	
N2	0.4777 (6)	−0.0108 (3)	0.1639 (3)	0.0737 (12)	
N3	0.8165 (6)	−0.0691 (4)	0.2100 (4)	0.0823 (13)	
O1	0.7880 (4)	0.0494 (3)	0.0418 (3)	0.0692 (9)	
O2	0.5682 (4)	−0.0941 (2)	0.0227 (2)	0.0574 (8)	
S1	1.11942 (18)	−0.11754 (13)	0.30794 (13)	0.0894 (5)	

### Atomic displacement parameters (Å^2^)

**Table:**

	*U*^11^	*U*^22^	*U*^33^	*U*^12^	*U*^13^	*U*^23^
Mn1	0.0669 (5)	0.0504 (5)	0.0598 (5)	0.0061 (3)	0.0259 (4)	−0.0011 (3)
C1	0.057 (3)	0.066 (3)	0.080 (3)	−0.005 (2)	0.005 (2)	0.012 (3)
C2	0.061 (3)	0.089 (4)	0.127 (6)	−0.007 (3)	0.023 (3)	0.022 (4)
C3	0.076 (4)	0.120 (7)	0.159 (8)	−0.037 (4)	0.010 (4)	0.042 (6)
C4	0.117 (7)	0.112 (7)	0.162 (9)	−0.061 (6)	−0.034 (6)	0.028 (6)
C5	0.133 (7)	0.085 (5)	0.123 (6)	−0.041 (5)	−0.029 (5)	0.002 (4)
C6	0.091 (4)	0.068 (3)	0.075 (4)	−0.012 (3)	−0.009 (3)	−0.001 (3)
C7	0.080 (3)	0.051 (3)	0.066 (3)	0.006 (2)	0.029 (3)	0.010 (2)
C8	0.130 (6)	0.064 (4)	0.071 (4)	0.009 (4)	−0.015 (4)	−0.016 (3)
C9	0.190 (9)	0.082 (5)	0.090 (5)	0.030 (5)	0.058 (5)	−0.015 (4)
C10	0.169 (8)	0.110 (6)	0.130 (6)	0.039 (6)	0.098 (6)	−0.011 (5)
C11	0.079 (3)	0.105 (5)	0.067 (3)	0.013 (3)	0.037 (3)	0.023 (3)
C12	0.061 (3)	0.079 (3)	0.066 (3)	0.000 (2)	0.018 (2)	0.020 (3)
C13	0.090 (4)	0.102 (5)	0.084 (4)	−0.019 (4)	0.025 (3)	0.030 (4)
C14	0.111 (5)	0.073 (4)	0.124 (6)	−0.021 (4)	0.017 (5)	0.035 (4)
C15	0.090 (4)	0.058 (3)	0.126 (6)	0.000 (3)	0.021 (4)	0.010 (3)
C16	0.070 (3)	0.053 (3)	0.093 (4)	0.003 (2)	0.021 (3)	0.005 (2)
C17	0.055 (2)	0.054 (2)	0.068 (3)	0.001 (2)	0.017 (2)	0.006 (2)
N1	0.114 (4)	0.061 (3)	0.061 (3)	0.016 (3)	0.020 (2)	−0.006 (2)
N2	0.085 (3)	0.079 (3)	0.070 (3)	0.015 (2)	0.043 (2)	0.006 (2)
N3	0.085 (3)	0.078 (3)	0.082 (3)	0.016 (3)	0.020 (3)	0.011 (2)
O1	0.069 (2)	0.060 (2)	0.084 (2)	−0.0027 (16)	0.0313 (18)	−0.0004 (17)
O2	0.0661 (19)	0.0491 (16)	0.0641 (18)	0.0026 (14)	0.0302 (15)	0.0026 (14)
S1	0.0697 (9)	0.0979 (12)	0.1010 (12)	0.0079 (8)	0.0256 (8)	0.0270 (9)

### Geometric parameters (Å, °)

**Table:**

Mn1—O1	1.874 (3)	C9—N1	1.469 (9)
Mn1—O2	1.902 (3)	C9—C10	1.552 (13)
Mn1—N1	1.979 (4)	C9—H9A	0.970
Mn1—N2	1.990 (5)	C9—H9B	0.970
Mn1—N3	2.181 (5)	C10—N2	1.503 (8)
Mn1—O2^i^	2.557 (3)	C10—H10A	0.970
C1—O1	1.339 (4)	C10—H10B	0.970
C1—C2	1.390	C11—N2	1.280 (8)
C1—C6	1.390	C11—C12	1.447 (7)
C2—C3	1.390	C11—H11	0.930
C2—H2	0.930	C12—C13	1.390
C3—C4	1.390	C12—C17	1.390
C3—H3	0.930	C13—C14	1.390
C4—C5	1.390	C13—H13	0.930
C4—H4	0.930	C14—C15	1.390
C5—C6	1.390	C14—H14	0.930
C5—H5	0.930	C15—C16	1.390
C6—C8	1.439 (9)	C15—H15	0.930
C7—N3	1.155 (7)	C16—C17	1.390
C7—S1	1.627 (6)	C16—H16	0.930
C8—N1	1.295 (9)	C17—O2	1.371 (4)
C8—H8	0.930		
			
O1—Mn1—O2	94.95 (14)	N1—C9—H9B	110.2
O1—Mn1—N1	92.1 (2)	C10—C9—H9B	110.2
O1—Mn1—N2	170.90 (17)	H9A—C9—H9B	108.5
O1—Mn1—N3	93.94 (18)	N2—C10—C9	104.1 (6)
O1—Mn1—O2^i^	88.62 (15)	N2—C10—H10A	110.9
O2—Mn1—N1	165.40 (19)	C9—C10—H10A	110.9
O2—Mn1—N2	89.51 (17)	N2—C10—H10B	110.9
O2—Mn1—N3	96.85 (17)	C9—C10—H10B	110.9
O2—Mn1—O2^i^	80.14 (14)	H10A—C10—H10B	109.0
N1—Mn1—N2	81.9 (2)	N2—C11—C12	124.6 (5)
N1—Mn1—N3	95.4 (2)	N2—C11—H11	117.7
N1—Mn1—O2^i^	87.29 (16)	C12—C11—H11	117.7
N2—Mn1—N3	93.4 (2)	C13—C12—C17	120.0
N2—Mn1—O2^i^	84.35 (17)	C13—C12—C11	117.6 (3)
N3—Mn1—O2^i^	176.24 (17)	C17—C12—C11	122.3 (3)
O1—C1—C2	117.4 (3)	C12—C13—C14	120.0
O1—C1—C6	122.4 (3)	C12—C13—H13	120.0
C2—C1—C6	120.0	C14—C13—H13	120.0
C3—C2—C1	120.0	C13—C14—C15	120.0
C3—C2—H2	120.0	C13—C14—H14	120.0
C1—C2—H2	120.0	C15—C14—H14	120.0
C2—C3—C4	120.0	C14—C15—C16	120.0
C2—C3—H3	120.0	C14—C15—H15	120.0
C4—C3—H3	120.0	C16—C15—H15	120.0
C5—C4—C3	120.0	C17—C16—C15	120.0
C5—C4—H4	120.0	C17—C16—H16	120.0
C3—C4—H4	120.0	C15—C16—H16	120.0
C4—C5—C6	120.0	O2—C17—C16	117.5 (2)
C4—C5—H5	120.0	O2—C17—C12	122.4 (2)
C6—C5—H5	120.0	C16—C17—C12	120.0
C5—C6—C1	120.0	C8—N1—C9	120.8 (6)
C5—C6—C8	116.3 (4)	C8—N1—Mn1	124.9 (4)
C1—C6—C8	123.6 (4)	C9—N1—Mn1	114.2 (4)
N3—C7—S1	177.1 (5)	C11—N2—C10	122.0 (6)
N1—C8—C6	126.1 (5)	C11—N2—Mn1	124.0 (4)
N1—C8—H8	117.0	C10—N2—Mn1	114.0 (5)
C6—C8—H8	117.0	C7—N3—Mn1	151.3 (5)
N1—C9—C10	107.5 (6)	C1—O1—Mn1	130.4 (3)
N1—C9—H9A	110.2	C17—O2—Mn1	120.8 (2)
C10—C9—H9A	110.2		

Symmetry codes: (i) −*x*+1, −*y*, −*z*.
